# Do truth-telling oaths improve honesty in crowd-working?

**DOI:** 10.1371/journal.pone.0244958

**Published:** 2021-01-15

**Authors:** Nicolas Jacquemet, Alexander G. James, Stéphane Luchini, James J. Murphy, Jason F. Shogren

**Affiliations:** 1 Paris School of Economics and Université Paris 1 Panthéon-Sorbonne, CES, Paris, France; 2 Department of Economics, University of Alaska Anchorage, Anchorage, Alaska, United States of America; 3 Aix-Marseille University, CNRS, EHESS, Centrale Marseille, Aix-Marseille School of Economics, Marseille, France; 4 Economic Science Institute, Chapman University, Orange, California, United States of America; 5 Department of Economics, University of Wyoming, Laramie, Wyoming, United States of America; Baylor University, UNITED STATES

## Abstract

This study explores whether an oath to honesty can reduce both shirking and lying among crowd-sourced internet workers. Using a classic coin-flip experiment, we first confirm that a substantial majority of Mechanical Turk workers both shirk and lie when reporting the number of heads flipped. We then demonstrate that lying can be reduced by first asking each worker to swear voluntarily on his or her honor to tell the truth in subsequent economic decisions. Even in this online, purely anonymous environment, the oath significantly reduced the percent of subjects telling “big” lies (by roughly 27%), but did not affect shirking. We also explore whether a truth-telling oath can be used as a screening device if implemented after decisions have been made. Conditional on flipping response, MTurk shirkers and workers who lied were significantly less likely to agree to an ex-post honesty oath. Our results suggest oaths may help elicit more truthful behavior, even in online crowd-sourced environments.

## 1 Introduction

Online labor markets have become increasingly popular. In social sciences research, for instance, crowd-work platforms like Amazon’s Mechanical Turk (MTurk) offer important advantages over the typical university student subject pool, including low cost, speed of data collection, and access to a more heterogeneous pool of participants [1–3], although far from being representative of the general population [4, 5]. Despite a more serious attrition problem [6], a large body of evidence shows consistency in behavior between MTurk workers and student subjects in multiple disciplines, including behavioral economics [7–9], psychology [10, 11], sociology [12], accounting [13], advertising [14] and political science [15]. Based on a large replication study [16], hypothesize this robustness might be related to the homogeneity of treatment effects measured in experiments in social sciences.

Nevertheless, there are concerns that the unique characteristics of online labor markets increase the potential for dishonest and unethical behavior. The workforce is anonymous and transient [17] and is typically unmonitored [18]. Because workers operate remotely in uncontrolled settings, online work can weaken social ties with employers [19] and has the potential for distractions such as cell phones [20] and multi-tasking [21]. Multiple studies have documented the prevalence of dishonest behavior on MTurk. This can manifest itself in multiple ways [see, *e.g*., 22, for a survey] including misrepresenting whether a worker meets the eligibility criteria for participating in a task [23], rushing through the task so quickly that it is not possible to properly perform the task [24] or shirking by not paying attention [25].

Our study provides direct evidence on dishonesty in online labor markets thanks to a variation of the coin-tossing game [26–28]: we asked MTurk workers to flip a coin 10 times and receive an additional 10 cents for each head observed. This design allows us to measure lying at the aggregate level, by comparing the distribution of outcomes to the theoretical truthful distribution. We complement this aggregate measure of lying with an individual measure of shirking, defined as answering the survey without performing the coin tossing task: we combine observed response times and external coin tossing data to classify workers as shirkers whenever their response was too quick to allow them to perform the required task. Consistent with other studies, our results show that dishonesty—both shirking and lying—is prevalent on MTurk: workers do lie, although not fully. Workers reported an average of 6.33 heads, which is significantly different from the expected mean of 5 if all workers were truthful, but is also much lower than the mean of 10 if all lied maximally. Shirking is widespread, with nearly half (42.6%) of workers who completed the coin-flip task at a rate that was physically impossible.

The open question we address herein is whether a non-financial honesty oath works to reduce dishonesty in crowd-working relationships. The solemn oath to honesty is an ancient and time-tested mechanism designed to eliminate misbehavior by asking a person to commit to the truth [29–32]. Using laboratory experiments, the oath has been shown to affect behavior in multiple contexts, including the reduction of hypothetical bias in non-market valuation [33–35], improving coordination in a strategic game with cheap talk [36] and increasing compliance in tax evasion games [37]. Both [38] and [39] directly test the effect of an oath on truth-telling in a lab setting with European university students, and show that the oath significantly reduced lying in the lab. We provide evidence on the effectiveness of a freely signed truth-telling oath in the field by assigning half the workers to an Oath treatment in which, before the coin-flipping task, they are offered the possibility to take a voluntary solemn oath to honesty.

Our results are threefold. First, while the oath slightly reduced shirking behavior (from 42% to 40%), this change was not significant (*p* = 0.170). Unless stated otherwise, the p-values provided in the text are associated with one-tailed t-tests. Second, the oath did, however, result in respondents spending almost 30 seconds more completing the survey (*p* = 0.002) which suggests that the oath may have induced them to be more thoughtful and accurate in their responses. Third, the oath caused a modest (4.2%) reduction in the average number of heads flipped (6.06 vs. 6.33 in the baseline treatment with no oath, *p* = 0.008) and a large (27%) reduction in the frequency of pay-off maximizing reports (12.9% vs 17.8%, *p* = 0.006). The oath thus causes workers to answer survey questions more thoughtfully and truthfully, and could be an effective and practical tool to elicit more accurate survey data.

Finally, we investigate whether a voluntary truth-telling oath can be used as a screening device to disentangle truthful answers from dishonesty. To that end, we implemented an ex-post truth-telling oath after the survey in the no-oath treatment. Our results show that both MTurk workers who reported flipping a large number of heads as well as those who did not carry out the coin-flipping task, were less likely to agree to the ex-post oath. Some workers voluntarily self-reported dishonest behavior when they were unexpectedly asked to take an oath attesting to the veracity of their completed work. Together, these methods can improve honesty in online labor markets, and data quality in online experiments.

## 2 Material and methods

Our empirical evidence comes from an online version of the coin-tossing game introduced by [26]. Our main treatment variable is a truth-telling oath adapted from [33]. The main outcomes of interest are the distribution of heads reported, and response times.

### 2.1 Survey implementation

The experiment was administered on Amazon’s Mechanical Turk, which is an online platform that connects employers (or “requesters”) with potential workers. The tasks (called “human intelligence tasks”, HITs) that MTurk workers (“MTurkers”) complete are typically simple and straightforward (*e.g*., answering a questionnaire) and can be completed privately and anonymously at any location. This platform has several advantages for the purpose of our study.

The first is representativeness. MTurkers operate in a naturally-occurring labor market and search for tasks with the goal of earning money. Our subjects answered a call soliciting participants. This stands in contrast with studies using a phone survey [*e.g*., 40] whose participants were not actively seeking opportunities to participate. MTurkers also tend to be more representative of the US population than in-person samples such as lab experiments [15].

The second is anonymity. MTurkers are only identified by a user id that cannot be linked to any personally identifying infor mation. Participants in lab studies, by contrast, are known to the experimenter even if decisions cannot be linked to an individual. Experimenters also usually have a direct, face-to-face interaction with subjects during check-in, reading of instructions and payment. While participants in phone surveys are typically anonymous, the researcher interacts directly with the subject during all stages, including decision-making, even though privacy and anonymity are maintained (although subjects do not necessarily understand it correctly if, for instance, they think the caller might have access to their name and address thanks to their phone information). The relationship between MTurkers and employers is fundamentally different because there is no direct personal contact. Employers post an ad describing the task, and if the worker accepts, then the task begins. Upon completion by the worker and acceptance by the employer, compensation is made.

Third, because all interactions are highly impersonal, Mturk weakens the connection between the researcher and the worker as compared to other implementations. This undermines the risk of demand effects—*i.e*., the will of subjects to comply with the experimenter request rather than providing their own preferred answer [41]. Fourth, for this same reason, lying behavior on Mturk is interesting on its own as impersonal market institutions like online labor markets have been hypothesized to foster self-interested non-cooperative behavior [42]. Lying to increase one’s earnings would be consistent with this hypothesis.

### 2.2 Content of the survey

The experiment was advertised on MTurk and MTurkers could only participate once. The only restriction on participation was to be at least 18 years old. After agreeing to the task, MTurkers were redirected to a Qualtrics survey. The first page thanked them for participating, and elicited informed consent to participate in an academic study. Respondents were informed they would be paid $1.00 for completing the survey, would have an opportunity to earn additional money during the survey, and that all of the information obtained would be kept anonymous and confidential.

#### Coin flipping task

The first screen of the survey reads “*Flip a coin 10 times. You will be paid a bonus of 10 cents per head that is flipped. How many heads did you flip?*”. While the monetary reward for lying is small, note that $1.00 amounts to roughly 30 minutes of work for a typical MTurker—the median wage on MTurk is just $2.00 per hour according to [43]. MTurkers were then provided a drop-down menu where they selected their answer from the list of integers between 0 and 10. Privacy and anonymity, combined with the bonus payment of 10 cents per reported head, create an incentive for MTurkers to report a larger number of heads than what they actually observed since there are no financial or social consequences for lying.

#### Demographic survey

After the coin-flipping task, respondents completed a survey which contained demographic questions and a subset of questions from the World Values Survey.

#### Consistency check

A common concern with Mechanical Turk is that workers are not thinking carefully about the questions being asked, or that automated programs (“bots”) that are designed to mimic human behavior contaminate the results. To address this concern, we follow [11] and add the following question about halfway through the survey “*We want to make sure you are not a robot. What is the number two plus the number three equal to?*”. Those MTurkers who gave an incorrect answer to this question are dropped from the analysis.

### 2.3 Treatment variables

#### Ex-ante oath

Respondents were randomly assigned to one of two treatments—Oath and NoOath. To avoid possible temporal bias, we released a batch of 200 HITs every two hours until the budget was exhausted. Upon agreeing to participate in the study, MTurkers in the Oath treatment were shown a screen that reads “*Before we begin, do you swear upon your honor to answer the following questions truthfully? (You will be allowed to continue with this survey regardless of your answer to this question)*”. In contrast with previous studies of the oath [e.g., 37, 38] MTurkers were not asked to sign the oath due to the online implementation of the procedure. Rather, they were asked to simply click “*yes*” or “*no*”. While agreeing to the oath was optional, only two MTurkers did not agree to this oath. Note, the oath was administered prior to MTurkers knowing anything about the task at hand, or the financial opportunity cost associated with being honest. Regardless of how MTurkers answered the oath question, they move to the next screen, which is the coin flipping task described above. The NoOath MTurkers were not presented with the oath screen, and instead went directly to the coin-flipping task. The content of the survey was otherwise exactly the same in both treatments.

#### Ex post oath

Immediately following the coin-flipping stage, MTurkers in the NoOath treatment were exposed to an ex-post oath that reads “*Do you swear upon your honor that the number of heads you reported flipping is truthful? (You will be paid according to the number of heads you reported flipping regardless of your answer to this question)*.” MTurkers in the Oath treatment were instead asked “*Did swearing upon your honor to tell the truth affect the number of heads you reported flipping?*”.

### 2.4 Measures: Lying and shirking

#### Lying

We define lying as intentionally making a false statement, which in this context means an MTurker misreported the actual number of heads observed after flipping the coin. A well-known feature of coin flip experiments is that lying cannot be observed at the individual level, since all decisions are made in private. Dishonesty can only be measured by comparing the aggregate outcomes to the truthful distribution—which requires a large enough sample size for the empirical distribution of draws to be close to the theoretical one. Participants are asked to perform 10 independent draws from a fair coin flip and to report the average of their draws: according to the central limit theorem, the distribution of this sample mean should be distributed normally, with an expected value equal to 5 and a variance equal to 1/4.

#### Shirking

We define shirking as the failure to perform the agreed upon task, *i.e*., not flipping the coin 10 times as instructed. While we do not observe respondents behavior during the survey, some (but not all) shirking can be detected at the individual level based on the amount of time an individual spent on the coin-flipping part of the survey. This response time is measured thanks to a feature embedded in the Qualtrics survey that records how long an individual spent on each page—*i.e*., the time in seconds elapsed between the page displays and the next page appears. This provides a reliable measure of the time spent on the task since MTurkers were required to answer each question to proceed to the next one, and were not allowed to go back and forth in the survey. Also note that, following standard practice, the survey does not mention the measurement of response times—which minimizes the risk that respondents manipulate the time they spend on the survey to pretend they performed the task.

To determine the minimum amount of time needed to complete this task, we asked 28 students in a large university class to flip a coin that had been provided to them 10 times as quickly as possible, count the number of heads, and enter the result online in the same way MTurkers in the experiment reported their answers. The fastest that any student completed the coin flipping task was 27 seconds, with a mean of 102 seconds. Based on this, we concluded that it was impossible to complete the task in less than 30 seconds (note that 30 seconds is a conservative estimate; in the classroom pilot, students already had a coin available and were prepared to flip before the timer started, whereas for the MTurkers, flipping time also included time spent getting a coin). One may be concerned that subjects did not have access to a coin while answering the survey. First note that subjects could have also “flipped” an online coin, using, *e.g*., Random.org, or the randomizer app on their smart phone. Such an alternative procedure is unlikely to save time as it requires three time-consuming steps. First a user enters an appropriate URL into the search bar (or accesses a mobile phone, unlocks it and opens the app). Second, the user makes a decision about the number of times a coin should be flipped. Third, the user must count the number of heads displayed, then enter the result into the Qualtrics survey. Assuming that the MTurker already knew of a coin-flipping website or had a randomizer app already installed on a phone, it is still highly unlikely that she would have been able to complete the task within 30 seconds. Second, we surveyed out-of-sample MTurkers and asked them. “*Do you have a coin within reach?’*’ Conditional on not having a coin within reach we then asked them, “*Could you get a coin within thirty seconds?*” Out of 454 responses, 335 (73.6%) reported having a coin in reach and 415 (91.2%) reported either having a coin in reach, or said they could get one in less than thirty seconds. Based on this threshold, we can identify those MTurkers who almost certainly did not complete the task (but we cannot identify those who certainly did complete it): we define a “quick” response as one that was completed in less than 30 seconds, and label those workers as “shirkers”. By contrast, a response that was completed in at least 30 seconds is defined as “slow”. Because the task was done in private, we have no way of knowing whether a “slow” MTurker actually performed the task—our measure based on quick responses thus provides a lower bound on shirking in the task.

### 2.5 Data

We collected data from 1, 410 MTurkers. Of these, we dropped the 43 (3%) MTurkers who failed to correctly answer the consistency check question (about what the sum of 2 + 3 equals). In addition, one MTurker who spent 1, 700 seconds answering the coin flipping question was dropped to minimize outlier bias when we examine flipping times. This leaves 1, 366 observations (681 in Oath and 685 in NoOath). Table 1 provides summary statistics on both treatments. Across the Oath and NoOath treatments, MTurkers were predominantly male (around 60%), white (63%) and physically located in the USA (82%). The average age was 35 (with a standard error equal to 10.7). Across all characteristics, MTurkers in the Oath and NoOath treatments were similar.

**Table 1:** Summary statistics.

	NoOath			Oath		
	Mean	Min	Max	Mean	Min	Max
**Survey outcomes**						
Heads Flipped	6.331	1	10	6.058	0	10
Flipping Time (s.)	56.87	2.25	971	57.9	2.179	987.057
Duration (s.)	214	53.7	2722	243	49	2057
Consistency check	0.974	0	1	0.964	0	1
**Individual characteristics**						
Male	0.627	0	1	0.593	0	1
Age	35.04	18	82	35.12	18	84
High Income	0.207	0	1	0.234	0	1
Low Income	0.405	0	1	0.417	0	1
US citizen	0.820	0	1	0.825	0	1
White	0.630	0	1	0.627	0	1
Black	0.056	0	1	0.061	0	1
Asian	0.055	0	1	0.057	0	1
Other race	0.202	0	1	0.255	0	1
No Religion	0.490	0	1	0.496	0	1
Hindu	0.124	0	1	0.117	0	1
Catholic	0.127	0	1	0.126	0	1
Protestant	0.162	0	1	0.165	0	1
Other religion	0.258	0	1	0.259	0	1
**Questions from the World value survey**						
Justified Benefits	2.023	0	10	2.020	0	10
Justified Transport	2.420	0	10	2.505	0	10
Justified Steal	1.394	0	10	1.350	0	10
Justified Taxes	1.943	0	10	1.998	0	10
Justified Bribe	1.643	0	10	1.625	0	10
Trust People	0.505	0	1	0.484	0	1
**Self-reported church attendance**						
No Church	0.550	0	1	0.552	0	1
Low Church	0.099	0	1	0.098	0	1
Med Church	0.109	0	1	0.117	0	1
High Church	0.035	0	1	0.036	0	1
*N*	684			682		

**Note**: Descriptive statistics on the main variables of interest in both treatments. See the S1 Appendix, Section A, for a detailed definition of the variables and the survey.

## 3 Results

### 3.1 Do MTurkers shirk and lie?

**Fig 1:**
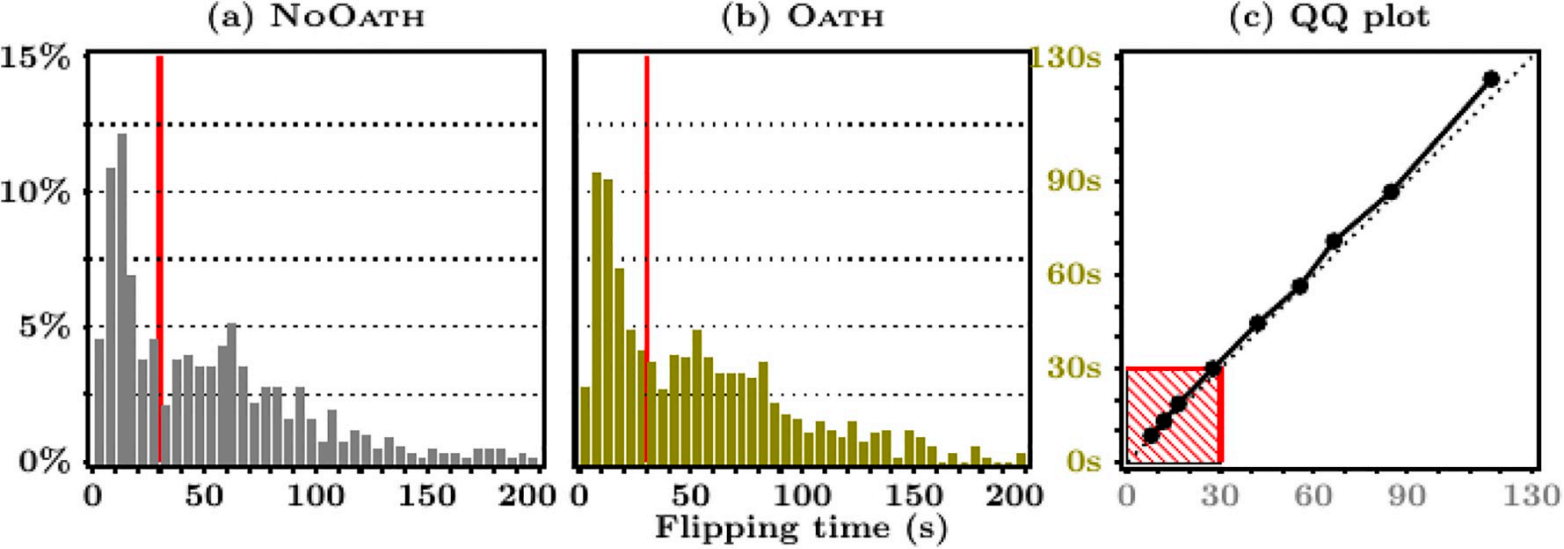
Flipping time distributions by treatment. **Note**. Panels (a) and (b) display the empirical distribution of flipping times, by treatment. A red vertical line is drawn at 30 seconds—the threshold defining shirkers. 35 workers who spent more than 200 seconds on the coin-flipping question were dropped to construct these figures. Panel (c) reports the QQ-plot of the quantiles of the NoOath flipping time distribution (on the x-axis) against the Oath one (on the y-axis).

We first focus on the NoOath treatment as a baseline to address this question of whether MTurkers shirk and/or lie. Fig 1 shows the distribution of flipping time by treatment (for display purposes, the figure omits those MTurkers who took more than 200 seconds). The vertical line displays the 30s threshold that distinguishes quick from slow responses. The data clearly indicate that, yes, a nontrivial number of MTurkers did not flip the coin as instructed, and did shirk: we observe that 42.6% (*N* = 292) of MTurkers completed the task in less than 30 seconds. This is comparable to the percent of inattentive MTurkers (42%) documented by [25].

**Fig 2:**
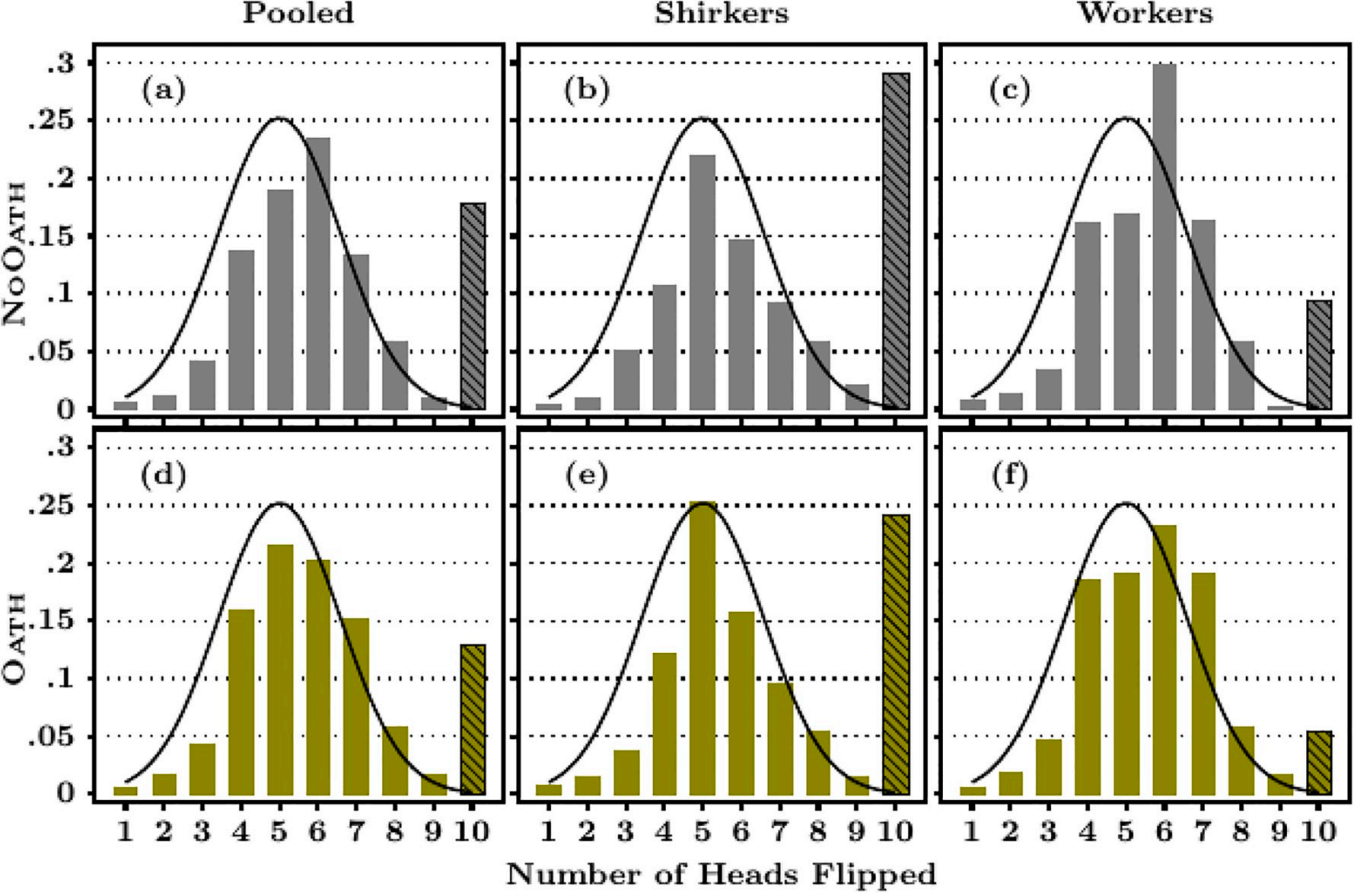
Heads flipped by flipping time and treatment. **Note**. Each figure provides the empirical distribution of heads reported, along with the theoretical truthful distribution. The shaded bars highlight the density of respondents who report having flipped 10 heads. The p-value from Shapiro-Wilk tests of the null hypothesis that each distribution is similar to the normal distribution is <.001 for both the overall distribution and the distribution conditional on the report being lower than 10.

Table 2 provides evidence on lying behavior based on the distribution of reported flips. Overall, MTurkers reported an average of 6.33 heads and we reject the null hypothesis that this is less than or equal to the expected mean of five if all reporting were truthful (*p* = 0.000). Fig 2 displays a more detailed comparison between reported outcomes and the truthful distribution. As shown in Panel (a), the modal response (*N* = 298, 21.8%) was six (a small lie if reported dishonestly), and 18% of MTurkers (*N* = 122) reported flipping 10 heads in a row (a “big lie”). This result is similar to [27] who find that 20% of subjects “lie to the fullest extent possible” in their die-rolling experiment. Note that the binomial probability of observing 10 heads is 0.1%, which implies that we should expect to observe this outcome no more than once in our sample if all MTurkers reported truthfully. If we put this extreme form of lying on the side, and disregard MTurkers who reported flipping 10 heads, the average number of reported heads flipped is 5.5, which is still statistically different from five (*p* = 0.000). We therefore conclude that, yes, on average MTurkers do lie. These lies come in two primary forms: some of these lies are plausible (*i.e*., reporting six) and others are implausible “big” lies that maximize the worker’s earnings (reporting 10).

**Table 2:** Reporting behavior by treatment, and response time.

	*N*	Avg Heads	Flipped 10	Shirker	Flipping Time	Duration
**Overall** (*N* = 1, 366)						
Oath	682	6.06	.129	.400	57.90	243.14
NoOath	684	6.33	.178	.426	56.87	214.23
*p*	—	.008	.006	.170	.389	.002
**Shirkers** (*N* = 565)						
Oath	273	6.49	.241	—	14.14	221
NoOath	292	6.79	.291	—	13.62	203
*p*	—	.072	.093	—	.196	.138
**Slow workers** (*N* = 801)						
Oath	409	5.76	.053	—	87.17	257
NoOath	392	5.98	.094	—	89.00	222
*p*	—	.041	.014	—	.637	.003

**Note**: The table reports, for the whole sample (*top part of the table*) and separately for “Shirkers” and “Slow workers” (*bottom part*), the average number of heads reported, the share of respondents who report having observed heads 10 times, the share who are classified as quick, the average flipping time and the average time spent on the remaining of the survey (both measured in seconds) in each treatment. The *p*-value corresponds to a one-tailed t-test of equality between oath and no oath values.

This conclusion that MTurkers lie is robust across both the shirkers (*i.e*., MTurkers who completed the task in under 30 seconds) and the slow workers for whom the time spent on the flipping task was sufficient for them to have possibly done the task. As shown in the bottom part of Table 2, shirkers reported more heads than the slow workers (6.79 vs 5.98, *p* = 0.000). Shirkers are also three times more likely to report observing 10 heads (29.1% vs 9.4%, *p* <.001, proportion test). Panels (b) and (c) of Fig 2 moreover show that while the modal responses for shirkers were five and 10, for slow workers the mode was six. Still, the mean number of heads reported by slow workers is 5.98 (which is significantly different from five, *p* <.001, and 9.4% of them reported 10 heads).

### 3.2 Does an oath reduce shirking and / or lying?

We now examine whether agreeing to a solemn oath causally affects reporting behavior. All respondents but 2 (0.29%) in the Oath treatment agreed to sign the oath. Table 2 shows the unconditional results. The average number of heads reported flipped by MTurkers in the Oath treatment was 6.06, which is 4.2% less than the number reported flipped by NoOath MTurkers (6.33, *p* = 0.008). That the mean exceeded five (*p* = 0.000) indicates that the oath is not a panacea for truth-telling. The oath also reduced the number of MTurkers who reported flipping 10 heads in a row by 27% (*p* = 0.006). In the Oath treatment, 88 MTurkers (12.9%) reported flipping 10 heads in a row whereas 122 (17.8%) of NoOath MTurkers did so.

The first column of Fig 2 gives the distribution of heads flipped for Oath and NoOath treatments. The “truthful distribution” is provided for comparison purposes (according to Shapiro-Wilk tests, the equality between the empirical and the theoretical distributions is rejected for all distributions). The distribution in the Oath treatment is significantly different from that for the NoOath treatment (*p* = 0.10, one-tailed Kolmogorov-Smirnov (KS) test). However, dropping MTurkers that reported flipping 10 heads, we cannot reject the null hypothesis that the oath had no effect (*p* = .713). This implies that the oath largely worked by decreasing the number of MTurkers that told big, obvious, lies which is consistent with the idea that telling big lies is more costly than telling small lies [44]. The S1 Appendix, Section C, shows that this change in behavior is unlikely to be due to changes in beliefs about the average behavior of others.

**Fig 3:**
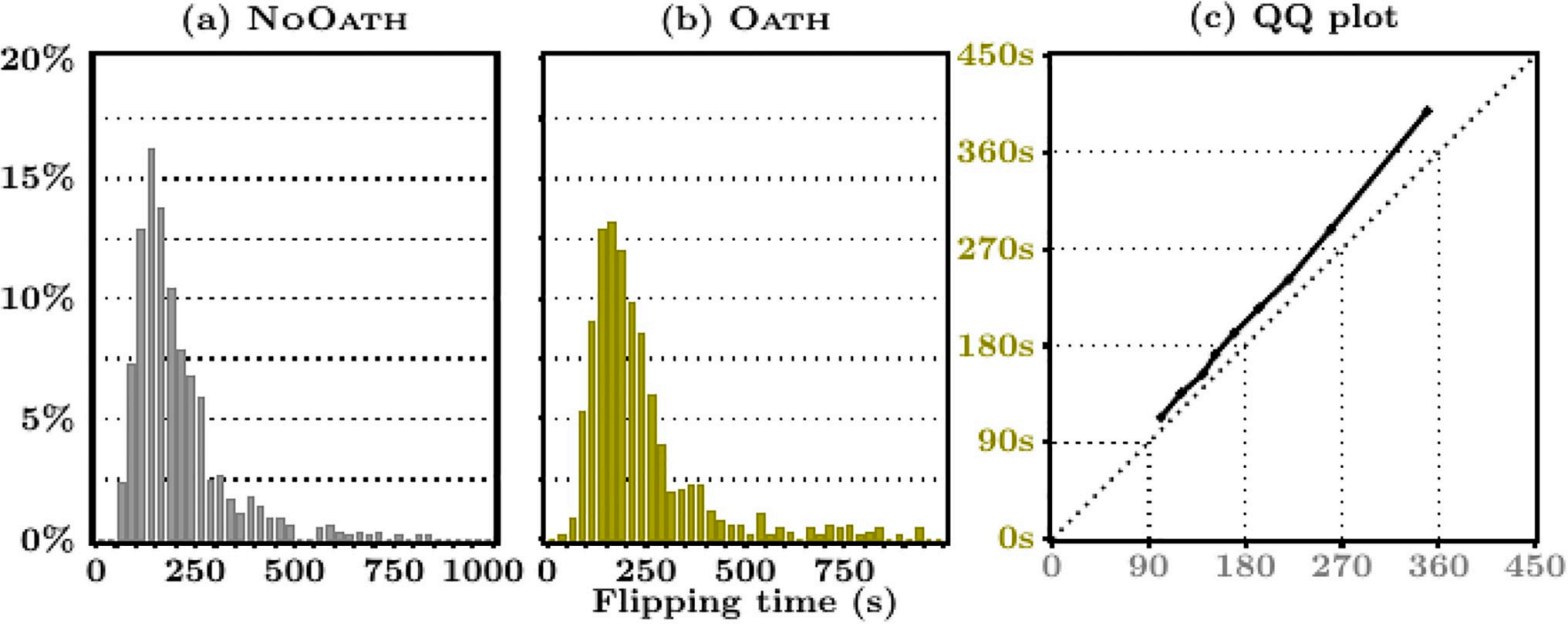
Empirical distribution of survey duration, by treatment. **Note**. The left-hand side figures report the empirical distribution of survey overall duration, net of flipping time. To ease readability, 13 workers for whom this duration is higher than 1000s were dropped to construct these figures. The figure on the right-hand side displays the QQ plot of the deciles of the net survey duration in the NoOath treatment (on the x-axis) against the Oath one (on the y-axis).

Table 2 also shows that the oath had little effect on the time MTurkers spent answering the coin-flipping question. Further, the oath had no effect on the probability an MTurker shirks (responds to the coin-flipping task in less than 30 seconds). This is confirmed by the empirical distribution of flipping times provided in Fig 1b, which is very similar to the one in the NoOath treatment. To ease the comparison, Fig 1c provides a QQ-plot of the two densities (which are statistically the same, *p* = 0.458, KS test). The relationship between the distribution of flipping time and the share of subjects reporting 10 heads in both treatments confirms the robustness of these conclusions to the choice of the shirking classification rule; see the S1 Appendix, Section B. However, we do observe that the oath induced MTurkers to spend approximately 30 additional seconds filling out the survey (net of the time spent on the coin-flipping task, see Fig 3). This amounts to roughly a 30/214 = 14% increase in survey duration. One speculative interpretation for these contrasting findings is that workers view their responses to survey questions as potentially consequential; their answers may directly influence any conclusions drawn from the study. In contrast, the coin-flipping task may be viewed as a time-consuming random number generator that can be costlessly avoided by strategically picking a number between zero and ten. While admittedly speculative, this theory is echoed by [25] who write that, “*For instance, if respondents to an attitudes survey fail to see the importance of the survey, they will not be attentive in their responses and will respond in a careless manner, yielding useless data*.” A potentially useful variant of the present study would be to ask subjects to carry out consequential tasks under oath.

Shirkers certainly did not carry out the task as requested whereas slow workers may have carried it out. We now examine the effect of the oath separately for these two groups of people. Table 2 shows that the oath was similarly effective at reducing the number of heads reported flipped by both shirkers and workers. According to Fig 2, the oath reduced the probability a shirker reports 10 heads, and increased the probability of reporting five heads. The oath had a similar effect for slow workers, but for this group the distribution is less bi-modal. However, we cannot reject the null hypothesis that the oath had no effect on the distribution of heads flipped for either shirkers or workers (*p* = .720, *p* = .370, KS test of equality between NoOath and Oath distributions in each group).

In sum, signing a truth-telling oath induced a dramatic decrease in big lies among both shirkers and slow workers. It however left unchanged the share of respondents who shirked.

### 3.3 Heterogeneous responses to the oath

We now turn to the role played by individual characteristics on both dishonesty and the response to the oath. Given the main lessons drawn in the previous section, we consider several outcomes to document dishonesty: the share of subjects who reported having flipped 10 heads in a row, the mean number of heads reported amongst subjects who report a number lower than 10 and the share of subjects who are classified as shirkers (based on flipping times). We also include the overall duration of the survey (net of flipping times) in the set of outcomes so as to asses the robustness of the effect of the oath on this variable.

Table 3 provides the results from Probit and OLS regression models. For each outcome variable, we first look at the heterogeneity in the likelihood of behaving dishonestly, based on regressions in the NoOath treatment, and then move to conditional estimates of the effect of the oath on pooled data from both treatments. The results show that age and gender are the two main sources of heterogeneity in behavior: being young or male increase the likelihood of both over-reporting the number of heads flipped and the likelihood of shirking, but increase the duration of the survey. This large gender difference confirms previous evidence on lying behavior [e.g., 45, 46]. We also find that US citizens were slightly less likely to tell big lies, and that lying was more widespread among Catholics and high-income individuals. The estimates of the effect of the oath conditional on observed heterogeneity confirm the main conclusions from the raw data: the oath significantly decreased the likelihood of reporting 10 heads, had a small and statistically insignificant effect on the mean number of heads reported in the remaining sub-sample, left unchanged the likelihood of shirking and significantly increased the overall duration of the survey.

**Table 3:** Conditional estimates of the effect of the Oath.

	(1) Report 10 heads		(2) Mean report cond. on *<* 10 heads		(3) Shirking		(4) Survey duration	
	NoOath	Pooled	NoOath	Pooled	NoOath	Pooled	NoOath	Pooled
Intercept	0.483	0.002	5.806***	5.717***	0.635	0.381	5.460***	5.199***
	0.415	0.993	0.000	0.000	0.140	0.142	0.000	0.000
Age	-0.023***	-0.019***	-0.009*	-0.011**	-0.019	-0.016***	0.002	0.003***
	0.000	0.000	0.078	0.010	0.000	0.000	0.295	0.004
Male	0.227*	0.188**	0.053	0.109	0.137	0.140*	-0.080**	-0.058**
	0.074	0.037	0.664	0.218	0.187	0.053	0.046	0.047
Asian	0.007	-0.342	-0.074	-0.043	0.190	-0.039	-0.092	0.054
	0.300	0.138	0.809	0.822	0.558	0.805	0.368	0.402
Black	0.171	-0.122	0.197	-0.049	0.132	-0.060	0.032	0.132*
	0.976	0.515	0.487	0.805	0.541	0.691	0.662	0.033
Other race	-0.509	0.071	-0.081	-0.370*	-0.180	-0.219	0.126	0.153**
	0.498	0.695	0.791	0.067	0.421	0.156	0.135	0.011
US Citizen	-0.904*	-0.420	0.003	0.238	-0.208	-0.084	-0.410***	-0.216**
	0.087	0.127	0.993	0.373	0.578	0.684	0.003	0.018
Catholic	0.333*	0.121	-0.005	0.111	0.415***	0.377***	0.066	0.070
	0.064	0.355	0.979	0.437	0.008	0.000	0.278	0.113
Protestant	0.088	0.181	-0.610	-0.346	-0.183	0.231	0.119	0.141*
	0.741	0.365	0.122	0.155	0.465	0.182	0.283	0.074
Hindu	0.063	-0.156	0.294	0.282	0.143	-0.056	-0.032	-0.048
	0.810	0.392	0.228	0.096	0.496	0.699	0.672	0.387
Other Religion	-0.087	0.029	-0.326	-0.293	-0.027	0.156	0.084	0.094*
	0.699	0.849	0.135	0.047	0.879	0.215	0.196	0.065
Low Income	0.070	-0.007	0.162	-0.054	-0.303***	-0.242***	0.068	0.075**
	0.573	0.936	0.232	0.573	0.004	0.001	0.105	0.016
High Income	-0.188	-0.050	0.543***	0.208	-0.131	-0.023	0.065	-0.011
	0.342	0.701	0.002	0.104	0.398	0.818	0.218	0.766
Oath	—	-0.181**	—	-0.050	—	-0.054	—	0.116***
	—	0.033	—	0.568	—	0.436	—	0.000

**Note**: (1) Probit regression on reporting 10 heads, (2) OLS with robust standard error on the mean number of heads reported conditional on not reporting ten, (3) Probit on being classified as a shirker based on flipping time, (4) OLS on the overall duration of the survey (net of flipping time). For each outcome variable (*N* = 684), the first column provides the regression in the NoOath treatment, the second column provides estimates on pooled data from both treatments (*N* = 1, 366). The reference individual is female, white, and atheist US citizen with medium income. For each variable (in row) the second line provides the p-value of the statistical significance of the estimate. Significance levels:

*10%,

**5%,

***1%.

This observed heterogeneity in dishonesty raises the question of heterogeneous responses to the oath. Coin flip experiments are not well-suited to investigate such heterogeneous responses, since truth-telling can only be observed at the aggregate level. This drastically lowers the statistical power of the analysis. We thus provide exploratory evidence on this question in Table 4, which disaggregates the three dishonesty outcomes across individual characteristics separately in each treatment (the sample size, reported in Table 1, varies across sub-groups as observed heterogeneity was not part of the randomization). In all sub-groups, and both treatments, we observe a large share of subjects who shirked and / or reported the maximum number of heads. Columns (3) and (6) report the mean number of heads reported among subjects in each sub-group whose report was lower than 10. The mean appears in bold whenever it is consistent with truth-telling behavior (*i.e*., the conditional mean is not different from five at the 10% level, the *p*-values are provided in the S1 Appendix, Section D). The results in the NoOath treatment provide a better understanding of the lying patterns in our sample. First, the average mean among subjects who did not lie maximally was generally close to five, suggesting that lies in these sub-populations were typically small. Second, reporting behavior was consistent with truth-telling for a few of these subgroups, in particular protestants [47], and non-US citizens. This last subgroup is also more likely to report 10 in Table 3 (this is true for 30% of them in the baseline, while the share is only 15% among US citizens), which suggests a strong self-selection on lying behavior in this sub-group: individuals who lied did it maximally, while others truthfully reported. The same applies to protestants, among whom 30% lie maximally while the remaining report truthfully. Interestingly, while the table confirms large differences in lying behavior according to gender (*e.g*., 12% of female respondents lie maximally, while 20% of male respondents do so) neither male nor female respondents who do not lie maximally truthfully report.

**Table 4:** Heterogeneity of responses.

	NoOath (*N* = 684)			Oath (*N* = 682)			Δ		
	(1)	(2)	(3)	(4)	(5)	(6)	(7)	(8)	(9)
	Shirking	10 heads	Cond.	Shirking	10 heads	Cond.	Shirking	10 heads	Cond.
	(%)	(%)	mean	(%)	(%)	mean	(%)	(%)	mean
**Individual covariates**									
30 years old or less	53.125	25.000	5.565	47.212	17.100	5.435	5.913*	7.900**	0.130
Between 31 and 37	38.776	16.327	5.604	35.897	11.795	5.715	2.878	4.532	-0.111
More than 38 years old	31.500	9.000	5.434	34.862	8.716	5.317	-3.362	0.284	0.117
Male	45.814	20.698	5.572	43.317	14.604	5.539	2.497	6.094**	0.033
Female	37.402	12.992	5.475	35.252	10.432	5.390	2.150	2.560	0.086
Race other	50.725	27.536	**5.170**	43.678	11.494	5.279	7.046	16.042**	-0.109
White	40.092	14.977	5.612	39.813	13.817	5.611	0.280	1.160	0.001
Asian	50.000	18.421	5.871	35.897	10.256	**5.429**	14.103	8.165	0.442
Black	38.462	20.513	5.548	30.952	11.905	**5.000**	7.509	8.608	0.548
US citizen	40.925	15.125	5.608	38.612	12.811	5.551	2.313	2.313	0.057
Not US citizen	50.820	30.328	**5.118**	46.667	13.333	**5.125**	4.153	16.995***	-0.007
Atheist	40.179	15.774	5.654	34.911	13.609	5.555	5.267	2.164	0.099
Catholic	54.023	21.839	5.691	50.000	11.628	5.711	4.023	10.211	-0.019
Protestant	47.619	30.952	**4.948**	53.086	16.049	**5.118**	-5.467	14.903*	-0.169
Hindu	40.541	13.514	5.573	35.398	7.965	5.481	5.142	5.549	0.092
Religion Other	39.548	13.559	5.464	38.983	10.734	5.373	0.565	2.825	0.091
Low Income	36.331	19.784	5.507	35.563	11.620	5.327	0.768	8.164**	0.180
Medium Income	49.186	18.241	5.414	43.682	14.440	5.616	5.503*	3.801	-0.202
High Income	40.404	11.111	5.943	42.149	12.397	5.519	-1.745	-1.286	0.424**
**Self-reported attitudes**									
Justified Benefits	48.579	24.289	5.553	49.872	16.624	5.571	-1.293	7.665**	-0.018
Unjustifed Benefits	35.017	9.428	5.513	26.804	7.904	5.362	8.213	1.524	0.151
Justified Transport	46.437	21.609	5.548	47.124	15.708	5.570	-0.687	5.901	-0.021
Unjustified Transport	36.145	11.245	5.511	26.087	7.391	5.310	10.058	3.854	0.201
Justified Steal	47.697	21.711	5.458	50.755	15.710	5.566	-3.058	6.001	-0.108
Unjustified Steal	38.684	14.737	5.590	29.915	10.256	5.397	8.770	4.480**	0.193*
Justified Taxes	49.180	22.404	5.518	50.667	16.800	5.587	-1.486	5.604*	-0.069
Unjustified Taxes	35.220	12.579	5.550	27.036	8.143	5.355	8.184	4.435*	0.196
Justified Bribe	48.765	23.148	5.482	53.890	18.156	5.489	-5.125*	4.993	-0.008
Unjustified Bribe	37.222	13.056	5.575	25.672	7.463	5.465	11.551	5.593*	0.111
Trust People	44.220	15.029	5.432	40.303	12.727	5.444	3.917	2.302	-0.012
Do not trust People	41.124	20.710	5.646	39.773	13.068	5.507	1.352	7.642**	0.139
No Church	38.727	16.711	5.675	33.777	12.766	5.567	4.950	3.945	0.108
Low Church	51.064	24.823	5.462	45.113	9.774	5.433	5.951	15.048***	0.029
Med Church	44.776	8.955	5.262	51.471	13.235	5.593	-6.694	-4.280	-0.331
High Church	44.444	18.182	5.284	48.571	17.143	5.115	-4.127	1.039	0.169

**Note**: Average observed value in both treatments of: (1) and (4) the share of shirkers, (2) and (3) the share of respondents who report 10, (3) and (6) the mean number of heads reported conditional on the report being lower than 10; by sub-groups defined in row. (7)-(9) report the observed difference between treatments, along with the statistical significance of the difference; in columns (3) and (5), all values are significantly different from 5 at the 10% unless they appear in bold (see the S1 Appendix, Section D, for all p-values). Significance levels:

* 10%,

** 5%,

*** 1%.

The right-hand side of Table 4 provides the outcomes observed in the Oath treatment along with the differences between treatments and their statistical significance (the *p*-values of all statistical tests are provided in the S1 Appendix, Section D). Both columns (4) and (7) confirm a negligible effect of the oath on the likelihood a subject shirked in all sub-groups. By contrast, the oath had a dramatic effect on lying behavior through a decrease in the likelihood of lying maximally by reporting 10 heads. This effect was stronger, and is statistically significant, in sub-populations in which such big lies were more widespread: young people, males, non US citizens and low income people. The oath also slightly reduced the share of protestants who lied maximally, while preserving the truthful reporting behavior of those who did not. Last, for both Asian and Black people who did not lie maximally, the mean number of heads became indistinguishable from truth-full reporting when under oath.

The bottom part of the table correlates the outcomes in both treatments with self-reported attitudes and church attendance. In the baseline, we observe that people who think it is often justified to cheat, steal, bribe, or fail to pay due taxes were more likely to report a high number of heads. We similarly find that people who trust others were less likely to report a high number of heads. The oath again had a stronger effect on the likelihood of lying maximally, and on the subgroups in which this share was the highest. We do not find any strong correlation between dishonesty and the frequency of church attendance in the NoOath treatment—which might be due to the heterogeneity of religious affiliations in our sample. The oath however had a significant effect on the likelihood of lying maximally on low church attendance people—the group in which this share was by far the highest in the baseline. The statistical tests commented on in the text do not account for multiple testing—the inflation in type I error probability due to the implementation of several independent tests on the same data. Table E in the S1 Appendix provides the results of a more conservative approach that adjusts the p-values to account for multiple testing. Based on this approach, the effect of the oath on non-US citizens and low-church attendance people remains significant at the 10% level.

### 3.4 Ex-post oath

Immediately after answering the coin-flipping question, NoOath MTurkers were asked “*Do you swear upon your honor that the number of heads you reported flipping is truthful*”. The acceptance rate is 90% (69 participants out of 685 decided not to sign). The average number of heads reported in this subgroup is 6.08, which is significantly greater than five (*p* = .000), but also significantly lower than the number of heads reported flipped by MTurkers who did not agree to the ex-post oath, equal to 8.50—a 30% decrease. The difference is again mainly driven by “big lies”. For example, the share of subjects who report having flipped 10 heads is 62.3% in the subgroup of respondents who refused to sign the ex-post oath, and 12.8% among the remaining NoOath participants (*p* <.001, proportion test). Still, we also observe a difference in ‘small lies’ as the average number of heads conditional on the report being lower than 10 is 6.15 in the first group, and 5.50 in the second one (*p* = 0.048). Interestingly, the screening implemented within the NoOath condition by an ex-post oath achieves outcomes that are similar to the ones observed in the entire population in Oath: both the proportion of Mturkers reporting 10 heads and the mean heads flip conditional on the report being lower than 10 are very similar: 12.9% vs 12.9% (*p* = 1, proportion test) and 5.50 vs 5.48 (*p* = .756).

Table 5 reports the results from Probit regressions of the willingness to sign the ex-post oath on the coin tossing task outcomes, with and without control variables. The results show that MTurkers who reported flipping a large number of heads were less likely to agree to the ex-post oath. This result is statistically significant and robust to conditioning on observed MTurker heterogeneity. We also find that MTurkers who reported flipping 10 heads in a row were less likely to agree to the ex-post oath—see columns (3) and (4). Interestingly, the effect of heads flipped remains negative after conditioning its effect on the indicator for flipping 10 heads as well as the indicator for shirking. This implies that even MTurkers who lied a little (did not report flipping 10 heads) were less likely to agree to the ex-post oath than people who reported more honest answers. Also, conditional on heads reported flipped, shirkers were less likely to agree to the ex-post oath—see column (6). This suggests that MTurkers who did not carry out the coin-flipping task may have viewed their behavior as dishonest, regardless of the answer they gave. Taken together, these results suggest that asking MTurkers to swear on their honor following the completion of a task may help identify shirkers and liars.

**Table 5:** Ex-post oath.

	Number of heads		Flipped 10 heads		Flipping Time	
	(1)	(2)	(3)	(4)	(5)	(6)
Heads	-0.276***	-0.280***			-0.149**	-0.165**
	(.038)	(.040)			(.071)	(.068)
Flipped 10			-1.28***	-1.316***	-0.688**	-0.489
			(.147)	(.157)	(.324)	(.315)
Flipping Time						-0.0001
						(.0009)
Shirker						-0.643***
						(.176)
Age		-0.001		-0.002	-0.002	-0.006
		(.006)		(.006)	(.006)	(.006)
Male		0.035		0.045	0.048	0.074
		(.155)		(.152)	(.154)	(.157)
White		-0.080		-0.110	-0.100	-0.078
		(.182)		(.183)	(.186)	(.188)
Black		-.059		-0.030	-0.053	-0.067
		(.362)		(.337)	(.351)	(.361)
Asian		-0.345		-0.422	-0.391	-0.388
		(.316)		(.315)	(.319)	(.326)
Constant	3.243***	3.384	1.664***	1.822***	2.699***	3.21***
	(.303)	(.492)	(.090)	(.327)	(.540)	(.538)
Pseudo *R*^2^	.172	.175	.170	.175	.187	.223

**Note**: Probit regressions on the likelihood that a respondent in NoOath agreed to the ex-post oath (*N* = 685).

## 4 Conclusion

We test whether workers on a crowd-working platform lie and shirk, and explore whether a solemn oath to be honest can reduce the prevalence of both. We asked roughly 1, 400 MTurk workers to flip a coin 10 times and report the number of heads they flipped. They were paid a bonus of 10 cents for each head reported flipped. In this environment, there is a clear and direct cost associated with telling the truth. Although we cannot tell whether individual workers told the truth, we can observe whether groups of people lied on average by comparing the distribution of reports to the underlying truthful distribution. Using response times, we are also able to identify shirkers individually—those MTurk workers who answered the coin-flipping question too quickly to have actually carried out the task.

We find that MTurk workers both lie (as measured by the distribution of heads reported flipped) and shirk (measured as the time spent on the coin flipping task). Offering respondents the possibility to sign a truth-telling oath reduces lying, but leaves shirking unchanged. Whereas workers reported to have flipped 6.33 heads on average in the baseline survey with no oath, workers under oath reported only 6.05 heads (a statistically significant reduction of 4.2%). While the magnitude of this change is small on average, the quantitative effect of the oath is more pronounced when examining “big” lies. MTurk workers who signed the oath were 27% less likely to report flipping 10 heads in a row (an event we should observe in less than 0.1% of the cases according to the true distribution). The oath also induced subjects to spend an additional 30 seconds answering the demographic survey (a 13.5% increase), suggesting the oath caused MTurk workers to answer questions more thoughtfully and carefully. Finally, we found that an ex-post oath (offered after decisions are made) is an efficient screening device: in the sub-population who agrees to sign such an oath, outcomes are behaviorally equivalent to the ones that arise in the entire population under an ex-ante oath.

It is possible that the failure of the oath to reduce shirking was because workers took an oath to honesty, rather than an oath to task (*i.e*., a commitment to actually perform the task as described). Future research should test whether an “oath to task” can reduce shirking. In addition, it is possible that one reason we observe a large amount of shirking on the coin-flipping task, but a significant effect of the oath on the amount of time spent on the survey, is because workers perceive the survey as meaningful or consequential, whereas reporting the number of heads flipped is viewed as less so. Future research could explore this conjecture further.

## Supporting information

S1 Appendix (PDF)
