# Correction: Do truth-telling oaths improve honesty in crowd-working?

**DOI:** 10.1371/journal.pone.0253997

**Published:** 2021-09-23

**Authors:** Nicolas Jacquemet, Alexander G. James, Stéphane Luchini, James J. Murphy, Jason F. Shogren

There are errors in the Author Contributions. The correct contributions are: All authors contributed equally to conceptualization, data curation, formal analysis, investigation and writing.
