# Supplementary material for: Do truth-telling oaths improve honesty in crowd-working?
Source: PLoS One. 2021 Jan 15;16(1):e0244958. doi: 10.1371/journal.pone.0244958 (PMC7810327; doi:10.1371/journal.pone.0244958)
Supplement: S1 Appendix — (PDF) [file pone.0244958.s001.pdf]

S1 Appendix:

A Variables Definition

Name	Survey Question #	Definition
Heads Flipped	Q3	Number of heads reported flipped, ranges from 0 to 10.
Male	Q8	= 1 for male workers.
Age	Q19	Reported age of worker.
White	Q28	= 1 for White workers.
Black	Q28	= 1 for Black workers.
Hispanic	Q28	= 1 for Hispanic workers.
Asian	Q28	= 1 for Asian workers.
Other	Q28	= 1 for non White, Black, Hispanic, Asian.
USA	Q25	= 1 for workers living in the U.S.A.
Justified Benefits[a]	Q57	"Is it ever justified to claim government benefits you are not entitled to?"
Justified Transport[a]	Q57	"Is it ever justified to avoid paying for public transport?"
Justified Steal[a]	Q57	"Is it ever justified to steal?"
Justified Taxes[a]	Q57	"Is it ever justified to cheat on taxes?"
Justified Bribe[a]	Q57	"Is it ever justified to accept a bribe?"
Trust People	Q41	Dummy variable =1 for workers who think "Most people can be trusted". = 0 for workers who think you "need to be very careful" in dealing with people.
SNAP Cheat	Q29	= 1 if more than 50% of food stamp recipients are believed to be "acting fraudulently, or otherwise cheating the U.S. welfare system"
Heads Guessed	Q30	"How many heads will the average worker report to have flipped?" Variable ranges from 0 to 10.
God	Q51	= 1 for workers who believe in god.
Hell	Q52	= 1 for workers who believe in hell.
No Church	Q49	= 1 for workers who report going to church "Never, practically never".
Low Church	Q49	= 1 for workers who report going to church "Once a month".
Med Church	Q49	= 1 for workers who report going to church "Once a week".
High Church	Q49	= 1 for workers who report going to church "More than once a week".
No Religion	Q26	= 1 for workers who "Do not belong to a denomination".
Hindu	Q26	= 1 for workers who are Hindu.
Muslim	Q26	= 1 for workers who are Muslim.
Jew	Q26	= 1 for workers who are Jewish.
Catholic	Q26	= 1 for workers who are Catholic.
Protestant	Q26	= 1 for workers who are Protestant.
High Income	Q21	= 1 for workers who report a household income greater than $99,000.
Low Income	Q21	= 1 for workers who report a household income less than $30,000.
Satisfied Financial[b]	Q43	"How satisfied are you with the financial situation of your household?"
Satisfied Generally[b]	Q39	"How satisfied are you with your life as a whole these days?"
Conservative	Q22	= 1 for workers who report 9 or 10 on a 10 point scale where 1=liberal and 10=conservative.
Liberal	Q22	= 1 for workers who report 1 or 2 on a 10 point scale where 1=liberal and 10=conservative.
Flipping Time	NA	Time to answer the coin flipping question.
Duration	NA	Total time to complete the survey, less flipping time.
2 + 3 Correct	Q60	"What is the number two plus the number three equal to?"

Note: The online survey that was administered is freely available at `https://www.protocols.io/view/lying-and-shirking-under-oath-bgw7jxhn`. Survey question numbers listed in the second column correspond to the oath treatment. "Other" includes Hawaiian and Pacific Islander, and non White, Hispanic, Black, and Asian.
a. Answers range from 0 (never justified) to 10 (always justified).
b. Answers range from 1 (not satisfied) to 10 (satisfied).

B Distribution of flipping times

As discussed in the text, the distribution of flipping time is not affected by signing the oath — resulting in a similar share of quick answers (which we use to classify respondents as shirkers) in both treatments. This is confirmed by the detailed data provided in Table A, which reports the quantiles of the flipping time distribution in each treatment.

Table A. Deciles of the flipping time distribution, by treatment

	Q_1 10%	Q_2 20%	Q_3 30%	Q_4 40%	Q_5 50%	Q_6 60%	Q_7 70%	Q_8 80%	Q_9 90%
**NoOath (s)**	7.3	11.6	16.3	25.7	40.8	54.6	65.5	84.1	116.5
**Oath (s)**	7.8	12.7	17.6	27.2	42.1	55.0	69.9	85.2	121.9
**Pooled (s)**	7.6	12.1	16.8	26.7	41.3	54.8	68.2	84.8	119.1

Table B reports the distribution of the share of subjects who report having flipped 10 heads in each treatment as a function of the distribution of flipping times. Two results emerge, which are in line with the conclusions drawn in the text based on the

empirical threshold defining shirkers: first, the share of subjects reporting 10 heads is much higher among respondents whose flipping time is short (Q_1 to Q_5, *i.e.*, subjects who respond in less than 40s). Second, the oath induces a significant decrease in this share at the bottom of the flipping time distribution.

Table B. Share of subjects reporting 10 heads as a function of flipping time

	Deciles of the flipping time distribution									
	Q_1	Q_2	Q_3	Q_4	Q_5	Q_6	Q_7	Q_8	Q_9	Q_{10}
**NoOath** (%)	26.7	34.7	22.9	29.9	26.1	4.5	4.9	10.0	5.5	12.3
**Oath** (%)	22.7	31.3	20.3	17.3	18.3	3.9	3.4	3.7	1.4	6.6

C Distribution of beliefs about the average behavior

Table C reports the observed distribution of beliefs about the average number of heads reported by other respondents in both treatments. The two distributions are statistically similar ($p = .853$, Kolmogorv-Smirnov test) so that the observed change in behavior induced by the oath cannot be attributed to a change in beliefs.

Table C. Distribution of beliefs about the average behavior

	Average report of others									
	1	2	3	4	5	6	7	8	9	10
**NoOath** (%)	0.15	0.44	1.61	4.09	33.77	15.79	16.67	13.89	5.26	8.33
**Oath** (%)	0.15	0.44	1.61	4.11	33.87	15.84	16.72	13.93	5.28	8.36

D Statistical tests supporting Table 4

Table D provides the p-values of the statistical tests reported in Table 4 in the text. The left-hand side reports t-tests of the null hypothesis that the mean report amongst subjects who do not report 10 is equal to 5. The right-hand side reports the p-values of the equality of outcomes between treatments based on proportion tests in the first and third columns, and two-samples t-tests in the second column.

Table E replicates all statistical tests using (1) approach to correct for multiple testing. Hypotheses are ordered according to their original p-value. Corrected p-values are then computed by weighting the original probabilities based on the total number of hypotheses tested and their rank.

Table D. Statistical tests supporting Table 4

	T test of mean equality to 5			Differences		
	NoOath	Oath		Shirking	10 heads	Mean
30 years old or less	0.000	0.000	30 years old or less	0.099	0.017	0.359
Between 31 and 37	0.000	0.000	Between 31 and 37	0.675	0.276	0.492
More than 38 years old	0.000	0.004	More than 38 years old	0.275	0.993	0.438
Male	0.000	0.000	Male	0.214	0.012	0.776
Female	0.000	0.000	Female	0.859	0.705	0.525
Race other	0.304	0.029	Race other	0.648	0.023	0.599
White	0.000	0.000	White	0.876	0.650	0.992
Asian	0.004	0.113	Asian	0.486	0.548	0.255
Black	0.077	1.000	Black	0.855	0.581	0.168
US citizen	0.000	0.000	US citizen	0.513	0.318	0.540
Not US citizen	0.529	0.415	Not US citizen	0.642	0.005	0.976
Atheist	0.000	0.000	Atheist	0.276	0.541	0.414
Catholic	0.000	0.000	Catholic	0.754	0.135	0.940
Protestant	0.810	0.515	Protestant	0.810	0.052	0.545
Hindu	0.000	0.002	Hindu	0.664	0.307	0.663
Religion Other	0.000	0.002	Religion Other	1.000	0.542	0.583
Low Income	0.000	0.001	Low Income	1.000	0.021	0.182
Medium Income	0.000	0.000	Medium Income	0.053	0.116	0.141
High Income	0.000	0.001	High Income	0.272	0.547	0.040
Justified Benefits	0.072	0.000	Justified Benefits	0.693	0.019	0.884
Unjustified Benefits	0.000	0.000	Unjustified Benefits	0.049	0.575	0.231
Justified Transport	0.110	0.000	Justified Transport	0.533	0.071	0.850
Unjustified Transport	0.000	0.003	Unjustified Transport	0.013	0.132	0.148
Justified Steal	0.000	0.000	Justified Steal	0.148	0.217	0.426
Unjustified Steal	0.000	0.000	Unjustified Steal	0.005	0.042	0.091
Justified Taxes	0.008	0.000	Justified Taxes	0.561	0.118	0.582
Unjustified Taxes	0.000	0.000	Unjustified Taxes	0.032	0.077	0.108
Justified Bribe	0.000	0.000	Justified Bribe	0.076	0.332	0.954
Unjustified Bribe	0.000	0.000	Unjustified Bribe	0.001	0.011	0.348
Trust People	0.000	0.000	Trust People	0.217	0.344	0.922
Do not trust people	0.000	0.000	Do not trust people	0.978	0.027	0.241
No Church	0.000	0.000	No Church	0.234	0.171	0.346
Low Church	0.001	0.002	Low Church	0.322	0.002	0.883
Med Church	0.149	0.008	Med Church	0.603	0.600	0.242
High Church	0.114	0.471	High Church	0.514	1.000	0.479

Table E. Replication of Table D with corrected p-values to account for multiple testing

	T test of mean equality to 5			Differences		
	NoOath	Oath		Shirking	10 heads	Mean
30 years old or less	0.000	0.000	30 years old or less	0.435	0.102	0.839
Between 31 and 37	0.000	0.000	Between 31 and 37	0.898	0.482	0.839
More than 38 years old	0.000	0.005	More than 38 years old	0.645	1.000	0.839
Male	0.000	0.000	Male	0.645	0.102	0.992
Female	0.000	0.000	Female	0.959	0.748	0.839
Race other	0.323	0.034	Race other	0.898	0.102	0.839
White	0.000	0.000	White	0.959	0.711	0.992
Asian	0.005	0.128	Asian	0.898	0.677	0.812
Black	0.090	1.000	Black	0.959	0.677	0.812
US citizen	0.000	0.000	US citizen	0.898	0.501	0.839
Not US citizen	0.544	0.453	Not US citizen	0.898	0.092	0.992
Atheist	0.000	0.000	Atheist	0.645	0.677	0.839
Catholic	0.000	0.000	Catholic	0.943	0.279	0.992
Protestant	0.810	0.530	Protestant	0.959	0.167	0.839
Hindu	0.000	0.003	Hindu	0.898	0.501	0.893
Religion Other	0.000	0.003	Religion Other	1.000	0.677	0.839
Low Income	0.000	0.001	Low Income	1.000	0.102	0.812
Medium Income	0.000	0.000	Medium Income	0.307	0.276	0.812
High Income	0.000	0.001	High Income	0.645	0.677	0.812
Justified Benefits	0.072	0.000	Justified Benefits	0.898	0.102	0.992
Unjustified Benefits	0.000	0.000	Unjustified Benefits	0.307	0.677	0.812
Justified Transport	0.110	0.000	Justified Transport	0.896	0.207	0.992
Unjustified Transport	0.000	0.004	Unjustified Transport	0.149	0.279	0.812
Justified Steal	0.000	0.000	Justified Steal	0.576	0.399	0.839
Unjustified Steal	0.000	0.000	Unjustified Steal	0.080	0.146	0.812
Justified Taxes	0.008	0.000	Justified Taxes	0.898	0.276	0.839
Unjustified Taxes	0.000	0.000	Unjustified Taxes	0.281	0.208	0.812
Justified Bribe	0.000	0.000	Justified Bribe	0.379	0.501	0.992
Unjustified Bribe	0.000	0.000	Unjustified Bribe	0.021	0.102	0.839
Trust People	0.000	0.000	Trust People	0.645	0.501	0.992
Do not trust people	0.000	0.000	Do not trust people	1.000	0.104	0.812
No Church	0.000	0.003	No Church	0.645	0.332	0.839
Low Church	0.002	0.002	Low Church	0.705	0.074	0.992
Med Church	0.163	0.010	Med Church	0.898	0.677	0.812
High Church	0.129	0.500	High Church	0.898	1.000	0.839

1. Benjamini Y, Hochberg Y. Controlling the False Discovery Rate: A Practical and Powerful Approach to Multiple Testing. Journal of the Royal Statistical Society Series B (Methodological). 1995;57(1):289–300.
